# The Effect of Superabsorbent Polymer on the Resilient and Plastic Strain Behavior of Cemented Soil under Traffic Load

**DOI:** 10.3390/polym14050929

**Published:** 2022-02-25

**Authors:** Di Dai, Jie Peng, Lanlan Bai, Gang Li, Hongmin Lin

**Affiliations:** 1 Key Laboratory of Ministry of Education for Geomechanics and Embankment Engineering, Hohai University, Nanjing 210098, China; daidiruiko@hhu.edu.cn (D.D.); linhm_hhu@163.com (H.L.); 2 JSTI Group, Nanjing 210019, China; bailanlan_jsti@163.com (L.B.); ligang_jsti@163.com (G.L.)

**Keywords:** dynamic triaxial test, SAP, traffic load, cement-stabilized soil, resilient property, accumulated plastic strain

## Abstract

In road construction, a large number of excavated soils need to be treated with stabilizers. The addition of superabsorbent polymer (SAP) can improve the road performance of these stabilized soils. In order to predict roadbed deformation, dynamic triaxial tests were carried out on cemented soil containing SAP to investigate its resilient and plastic strain behavior. The effects of SAP content, cyclic stress ratio, and loading frequency on cement-stabilized soils with SAP were analyzed combined with the number of cycles. This study demonstrates how these influencing factors effect the resilient strain, dynamic elastic modulus, and accumulated plastic strain, which are crucial to better understanding the strain behavior of cement-stabilized soil with SAP. The results show that SAP can significantly improve the brittle failure characteristics and dynamic strength of cement-stabilized soil. Soil with higher SAP content possesses smaller accumulated plastic strain; with the increase in the cyclic stress ratio, the dynamic elastic modulus decreases significantly, whereas the accumulated plastic strain has the opposite trend. In addition, the lower frequency produces larger cumulative axial strain.

## 1. Introduction

In order to promote the process of urbanization, vigorously developing urban expressways, urban light rails, and metros have become key infrastructure projects. However, there is often a large amount of excavated soil to be processed in the construction of roads and tunnels. Most of these excavated soils belong to soft soil. Soft soil is a regional special soil with high water content, a large pore ratio, high compressibility, low permeability, and high sensitivity [1,2,3]. Especially along the lower reaches of the Yangtze River in China, the widespread distribution of soft soil has become a factor restricting the process of urbanization. In addition, the world is in the context of sustainable development, so the reasonable handling of these excavated soils has become an urgent engineering and environmental problem.

In field construction, the method of mixing stabilizer is often used to treat excavated soil. The method of mixing stabilizer is to use ordinary Portland cement and other cementing materials to treat soil, which can effectively improve the unconfined compressive strength of soil and reduce its compressibility [4,5]. Adding ordinary Portland cement causes a hydration reaction in the soil to form cementitious materials and cement soil particles and fill the pores in the soil. Some scholars believe that with an increase in the water–cement ratio in the soil, the porosity of the soil will also increase, leading to a relatively loose cementation structure in the soil and showing a decrease in strength. Therefore, in order to ensure the strength of cemented soil, it is often necessary to add a large amount of ordinary Portland cement or other traditional cementing materials in the treatment of soil with high moisture content [6,7]. However, after the extensive use of ordinary Portland cement, cemented soil has dry shrinkage similar to concrete materials, and its durability is poor in dry–wet cycling conditions [5,8].

In order to improve the mechanical properties of cemented soil, fly ash, slag, and organic polymers are mixed in the cement-mixing method. Among them, organic polymers are often used to treat sand to refine the loose structure and improve the strength of soil [9,10,11]. It is worth noting that the water consumption of these cementing materials is limited, and the main way to improve the strength of excavated soil is to reduce its water content. Specifically, organic polymers are mainly used in sand and gravel, which are relatively dry materials. Therefore, in order to treat excavated soil with relatively higher water content, a class of superabsorbent polymers (SAP) has attracted the attention of researchers. SAP is a water-absorbing agent with excellent performance. It is often used as a water-retaining agent and water-saving agent in agriculture to improve soil performance [12,13]. In engineering, it is often used to improve self-drying properties of concrete and cement mortar [14,15,16]. Test results show that SAP has a positive effect on the unconfined strength and compressibility of soil with higher moisture content (such as dredged silt and excavated soft soil) [2,6,7,17]. Furthermore, SAP can improve the dry shrinkage of cemented soil and the durability under dry–wet cycling [18,19,20]. Therefore, it is generally believed that SAP can improve the static characteristics of cemented soil, but the dynamic characteristics need to be considered when SAP is combined with cement to treat excavated soil and backfilling as subgrade.

The dynamic characteristics of soil under traffic load can be used to predict subgrade deformation. In modern cities, first-class highways and subway tunnels often coincide at different depths, so when considering the subgrade soil of first-class highways, not only should the traffic load on the road surface be considered, but also the traffic load in the tunnel. When the subway passes through the tunnel, the additional normal vertical stress may far exceed the stress in other directions, resulting in a deviation between the soil dynamic response under traffic load and that under typical seismic load [21,22]. Previous researchers have explored the dynamic characteristics of subgrade soil. Cui et al. [23] studied the permanent deformation of soil under long-term cyclic load and established corresponding models to predict permanent deformation. Wichtmann et al. [22] believed that with the increase in cycles, the continuously generated plastic strain may change the dynamic parameters of the soil. At present, it is generally considered that the constant amplitude dynamic load can be used to obtain dynamic indexes such as the dynamic strain, dynamic elastic modulus, and damping ratio of soil. Guo et al. [24] studied the changes in the dynamic strain and dynamic elastic modulus of undisturbed soft soil through dynamic triaxial tests. Lei et al. [25] studied the effects of loading frequency and dynamic stress increment on long-term deformation behavior and the dynamic modulus of super soft soil by dynamic triaxial test. The above results show that the dynamic elastic modulus of soft soil decreases rapidly and sharply at the beginning, and reaches a stable value after several cycles, which may lead to the soil not meeting the design requirements of subgrade. Therefore, it is necessary to further study the dynamic characteristics of cemented soil. Scholars have studied the change rules and influencing factors of critical dynamic stress, cumulative plastic deformation, elastic deformation, and the resilient modulus. In terms of the dynamic strain of cemented soil, it is generally believed that it undergoes recoverable (resilient) and permanent (plastic) strains under traffic load [26,27,28]. Furthermore, scholars studied the response process of cemented soil and other cementing materials to cyclic loading. When the cyclic stress ratio is less than the oscillation limit of the soil, the plastic strain of the soil accumulates rapidly at the start of loading. With the increase in the number of cycles, the increased rate of plastic strain gradually decreases and approaches zero, and the final deformation tends to be completely resilient. When the cyclic stress ratio is greater than the oscillation limit of the soil but less than the creep limit, the plastic strain accumulation rate is slow, the soil needs to experience more cycles to tend to be fully resilient, and the accumulated plastic strain changes greatly. When the cyclic stress ratio is greater than the creep limit of the soil, the plastic strain accumulates rapidly, and the soil loses its strength under fewer cycles [29,30,31]. In addition, scholars also summarized empirical formulas for predicting the dynamic elastic modulus and damping ratio [32,33,34]. It is generally believed that the dynamic characteristics of cemented soil are significantly improved compared to soft soil, but its critical dynamic stress decreases [35]. Some scholars believe that in the middle and high strain range, with an increase in cement content, the soil stiffness decreases rapidly (the dynamic modulus increases rapidly), resulting in a change in the soil from tough to brittle, which leads to a decrease in dynamic strength and brittle failure [36]. In addition, some scholars have studied the influence of SAP on the mechanical properties of clay and found that SAP has a weakening effect on the dynamic modulus of clay [37]. However, there are few reports on the effect of SAP on the dynamic strain and dynamic elastic modulus of cemented soil under traffic load. Before predicting the deformation behavior of subgrade, it is necessary to accurately understand the stress distribution, and reasonable dynamic parameters can ensure the consistency of numerical simulation and measurement results. Therefore, it is very important to determine the dynamic characteristics of cemented soil with SAP under traffic load reasonably.

The main purpose of this study is to study the variation of dynamic strain and dynamic elastic modulus of cemented soil and cemented soil with SAP under different increasing dynamic stress conditions so as to determine the influence of SAP on the dynamic characteristics of cemented soil under traffic load. In this study, the effects of SAP content, cyclic stress ratio, and vibration frequency on the elastic strain of cemented soil were first studied through a dynamic triaxial test to determine the effect of SAP on the dynamic modulus of cemented soil. Then, the effects of SAP content, cyclic stress ratio, and vibration frequency on the plastic strain of cemented soil were analyzed to determine the effect of SAP on the dynamic strain of cemented soil.

## 2. Test Introduction

### 2.1. Materials and Sample Preparation

Soil samples were collected from the test section of highway construction in the lower reaches of the Yangtze River in Jiangsu Province, China. The basic physical properties of the excavated soil are shown in Table 1.

**Figure 1:**
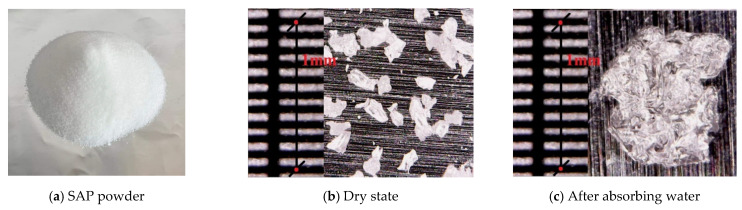
Comparison of SAP before and after water absorption.

The chemical composition of the ordinary Portland cement used in this study is shown in Table 2. The SAP used in this study belongs to polyacrylate absorbent resin. In the dry state, the diameter of SAP particles is about 100–150 μm, and the water absorption per gram of SAP is about 60 g (as shown in Figure 1).

In this study, samples were prepared via remolding method. The air-dried excavated soil was broken with wooden hammers and then a predetermined amount of water was mixed with the excavated soil. After standing in the sealed tank for 24 h, the cement powder and SAP particles were added to the excavated soil. Then the mixture was stirred for 5–10 min to meet the requirements of uniformity. The mixed sample was layered and compacted to make a cylindrical sample with a height of 76 mm and a diameter of 38 mm. In order to ensure sample saturation, after sample preparation, the sample was placed in a standard curing chamber (temperature: 20 ℃ ± 2 ℃, humidity: more than 95%) for 1 day to make the samples had a certain structure, then placed in a vacuum barrel for saturation by vacuumizing. After saturation, the samples were placed in the standard curing chamber for seven days. After curing, they were vacuumized again for saturation, and then the back stress of the samples on the instrument was increased to saturate them until the B-value reached 0.98.

### 2.2. Test Equipment

The test equipment used in this study is a servo motor-controlled dynamic triaxial test system (GDS-DYNTTS 2 Hz/10 kN, GDS Instruments, Hampshire, UK) equipped with automatic numerical control and a data acquisition system (GDSLAB). The displacement accuracy is 35 μm/50 mm (equivalent to 0.07% of the range), the displacement resolution is 0.208 μm, the axial force resolution is 16 bits, and the maximum number of data points per period is 1000. The device allows for long-term cyclic loading tests at loading frequencies of less than 2 Hz.

### 2.3. Test Conditions and Scheme

The appropriate loading mode, loading frequency, cycle number, cyclic stress ratio, and effective stress needs to be chosen for traffic load. Sinusoidal wave loading was selected as the loading method, because sinusoidal waves have a more obvious impact on soil deformation than semi-sinusoidal waves and are more suitable for the coincidence of subway tunnels and first-level highways [38,39]. In terms of loading frequency, the selection was 1 Hz, which is regarded as the frequency chosen most frequently on behalf of real traffic [24]. It is generally considered that 5000–6000 cycles are medium-term and long-term loads [40,41]. A total of 6000 cycles was selected for this experiment (50 data points were recorded in each cycle), unless the axial strain of the sample reached 10% in advance or the sample was obviously damaged so the same amount of dynamic stress could not be applied. It is generally considered that 0.18–0.28 is the cyclic stress ratio of traffic load [38]. Considering the need to test SAP’s improvement of cemented soil, the cyclic stress ratios of this test were 0.2, 0.4, and 0.6, and the calculation formula was as shown in Equation (1):

(1) $$CSR=\frac{\sigma_{d}}{2\sigma_{c}}$$

Among them, CSR is the cyclic stress ratio, $\sigma_{d}$ is the dynamic stress increase, and $\sigma_{c}$ is the effective stress. As the depth of backfill after excavation soil treatment was 5–15 m, the effective stress was selected as 100 and 200 kPa. The specific test plan is shown in Table 3.

### 2.4. Dynamic Parameter Definition

The dynamic elastic modulus is a secant modulus describing the dynamic stress–strain relationship under cyclic load, which plays a very important role in predicting the condition of subgrade soil. Formula (2) can be used to calculate it:

(2) $$E_{d}=\frac{\sigma_{dmax}-\sigma_{dmin}}{\varepsilon_{dmax}-\varepsilon_{dmin}}$$

**Figure 2:**
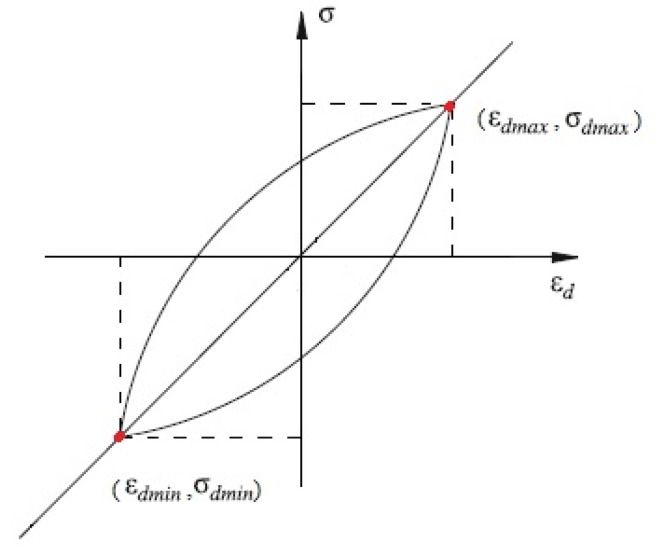
Schematic diagram of calculating the dynamic elastic modulus.

Among them, $E_{d}$ is the dynamic elastic modulus; $\sigma_{dmax}$ and $\sigma_{dmin}$ are the maximum and minimum values of the dynamic stress, respectively; and $\varepsilon_{dmax}$ and $\varepsilon_{dmin}$ are the corresponding dynamic strains, as shown in Figure 2.

## 3. Results and Discussion

### 3.1. Dynamic Strain Behavior of Soil

**Figure 3:**
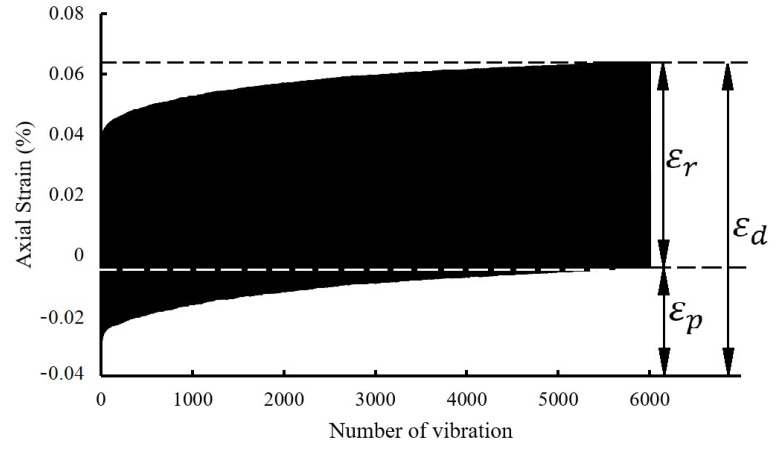
Curve of deformation characteristics on cemented soil with SAP.

The deformation of soil under traffic load is one of the characteristics that must be paid attention to. The axial accumulation data of symbol C4S30 under the conditions of effective confining pressure of 100 kPa, loading frequency of 1 Hz, and CSR of 0.2 are plotted in Figure 3. In the vibration process, the total dynamic strain $\varepsilon_{d}$ consists of two parts, namely, elastic strain $\varepsilon_{r}$ and plastic strain $\varepsilon_{p}$. The calculation relationship of them is described in Figure 3. The specific calculation formula is shown in Formula (3):

(3) $$\varepsilon_{d}=\varepsilon_{r}+\varepsilon_{p}$$

It can be seen from Figure 3 that the dynamic strain curve of soil generally rose from steep to close, which indicates that the dynamic strain included two stages, namely, the rapid growth stage and the stable increase stage. In the stage of rapid growth, a large amount of strain was rapidly generated in the soil, and then the rate of strain accumulation gradually slowed down and the maximum dynamic strain was accumulated in this stage. When the cumulative strain rate tended to be stable and at a low level, the soil entered the stable growth stage. At this stage, the cumulative strain rate of the soil was relatively stable, and the dynamic strain of the soil increased slowly. Combined with the actual working conditions, the deformation of subgrade soil and soil around the tunnel mostly occurred at the early stage of the road or subway operation. With the increase in operation time, the soil gradually became denser. The analysis on this aspect is discussed in the subsequent section.

In terms of plastic strain, it was consistent with the development trend of the total dynamic strain. The plastic strain increased rapidly at first, and then with the increase in vibration time, the growth rate of the plastic strain decreased gradually and most of the plastic strain occurred in the first 1500 vibrations. In terms of elastic strain, there was a similar trend, but the elastic strain tended to be stable after about 200 vibrations—that is, the change rate of the elastic strain tended to zero after 200 vibrations.

Due to the limitation of the vibration number, the total dynamic strain was not observed as tending to a stable value. However, it is generally believed that after the stable stage, the soil enters the damply increasing stage [29,42], in which the increasing rate of dynamic strain gradually decreases and negative growth may occur. This further indicates that the dynamic strain of soil subjected to traffic load is mainly concentrated in the first two stages.

### 3.2. Resilient Strain Behavior

#### 3.2.1. Resilient Strain under Different SAP Contents

**Figure 4:**
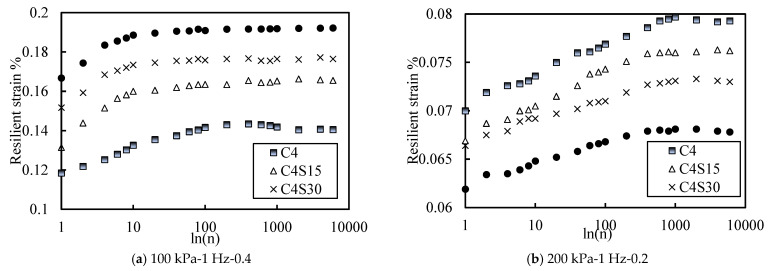
Curve of resilient strain with different contents of SAP.

Based on the preliminary exploration of SAP improving cemented soil and the summary of previous studies, it is generally believed that SAP mainly plays a filling role in soil [43,44,45]. These results indicate that different SAP content could change the size and distribution of pores in soil, leading to the rearrangement of the soil structure. Figure 4a shows the relationship between elastic strain and vibrations of soil samples with different SAP contents under the conditions of an effective confining pressure of 100 kPa, a CSR of 0.4, and a loading frequency of 1 Hz. Similar variation rules applied to soil samples with an effective confining pressure of 100 kPa. At the beginning of the vibrations, the elastic strain of the soil sample increased by a large increment. In the first 200 vibrations, this increment slowed down gradually with the increase in the vibration number, and after 400 vibrations, the elastic strain tended to a stable value. The overall elastic strain varied from 0.12 to 0.19%. By comparing each curve, it was not difficult to find that the elastic strain of the soil increased with the increase in SAP content, but the curve of samples containing SAP tended to level off after about 20 vibrations. However, the curve of samples without SAP approached the smooth value after 200 vibrations. These results indicate that SAP weakened the instantaneous deformation resistance of the cemented soil, but the deformation tended to be stable in a very short time, which made the soil enter the stable increase stage more quickly. This may be because SAP plays a filling role in the soil. The higher the content of SAP is, the easier it is to lose the original cohesion between the soil particles. In addition, SAP particles are hydrophilic groups in the soil, and it is difficult to have new cohesion with the soil particles [43], which leads to the macroscopic performance of soil containing SAP, decreasing its transient deformation resistance. Furthermore, SAP can fill the pores in the soil body, which lessens the number of compressible pores in the soil body after vibration. Macroscopically, soil containing SAP can enter the stable increase stage faster.

Figure 4b shows the relationship curve between elastic strain and vibrations of soil samples with different SAP content under the condition of an effective confining pressure of 200 kPa, a CSR of 0.2, and a loading frequency of 1 Hz. Unlike the trend under the effective confining pressure of 100 kPa, when the effective confining pressure was 200 kPa, the larger the SAP content was and the smaller the elastic strain was. In other words, under the condition of high effective stress, SAP could improve the instantaneous anti-deformation resistance of soil and make the soil enter the stable increase stage faster. This may be a higher effective confining pressure, so the pores in the soil are further reduced and the soil enters the compressive yield state, which makes the dominant factor of deformation resistance become the pore size in the soil. Within the content controlled by the test, the higher the SAP content was, the smaller the pore size of the soil was. Therefore, the soil sample with high SAP content had better instantaneous anti-deformation ability and could enter the stable increase stage faster.

**Figure 5:**
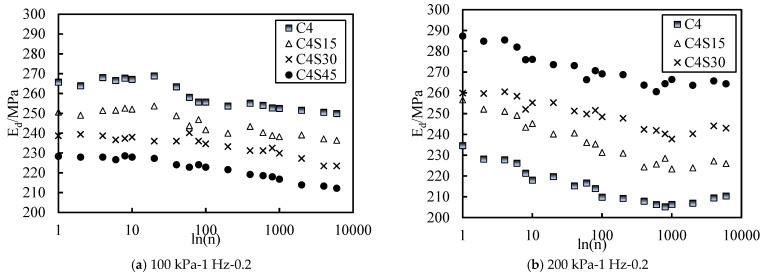
Curve of dynamic elastic modulus with different contents of SAP.

Based on Formula (2), the data of the dynamic elastic modulus $E_{d}$ are plotted in Figure 5. The trend of the dynamic elastic modulus $E_{d}$ was opposite to that of the elastic strain, but because the dynamic modulus $E_{d}$ is related to the reciprocal of the strain, the trend of the actual dynamic modulus $E_{d}$ was consistent with that of the elastic strain. It is easy to see from the overall observation that the variation range of the dynamic modulus was relatively concentrated. When the effective stress was high, the addition of SAP improved the dynamic elastic modulus of the soil. When the effective stress was low, SAP reduced the dynamic elastic modulus of the soil, which is worth our attention. When the effective stress was 100 kPa, usually the corresponding buried depth was 5–6 m, which is mainly used as subgrade soil. Therefore, it is necessary to consider whether the addition of SAP will cause the soil to fail to meet the requirements of the dynamic elastic modulus in subgrade filling. Combined with the American Asphalt Association (AI) design method and AASHTO-T-292-1997, it is known that the value of the dynamic elastic modulus of subgrade soil should not be less than 10.3 CBR. According to the provisions of JTG D30-2015 in China, the CBR value of the subgrade soil of first-grade highways should be greater than 8, and the dynamic elastic modulus of subgrade soil should not be less than 82.4 MPa after conversion. The data presented in Figure 5 are far beyond the design requirements. Combined with previous studies, it is known that the addition of cement increases the dynamic elastic modulus of soil, which is due to the formation of a cement bond between soil particles, with a denser structure. Under the same dynamic load, the dense structure can consume more kinetic energy, which is characterized by a large dynamic elastic modulus [46]. SAP is a hydrophilic group in the soil [47]. Some studies have shown that due to the lubrication effect of water, the dynamic elastic modulus of cemented soil decreases with the increase in water content [46,48], which explains the influence of SAP on the dynamic elastic modulus of cemented soil under low effective stress conditions. With the increase in effective stress, the soil obtains greater compaction energy, which increases the unit weight of soil and makes the structure denser. Therefore, the main factor affecting the dynamic elastic modulus of soil at this time is compaction energy [37,49]. Due to the filling effect of SAP, it can play a buffer role when bearing greater compaction energy, so the soil can consume more kinetic energy when subjected to dynamic load. Therefore, the sample containing SAP made the soil sample more dense after absorbing more compaction energy, which is consistent with the previous conclusion about the influence of SAP on the compactness of cemented soil [44]. Horpibulsuk et al. [50] also achieved similar findings when studying the optimal water content of cemented materials.

In conclusion, under the condition of low effective stress, the dynamic elastic modulus of cemented soil decreases with the increase in SAP content, but it can still meet the design requirements of subgrade soil. SAP can improve the dynamic elastic modulus of cemented soil under high effective stress. Therefore, SAP can be added according to the needs of the project, and its influence on the dynamic elastic modulus of soil can be ignored in practical engineering.

#### 3.2.2. Resilient Strain under Different CSR

**Figure 6:**
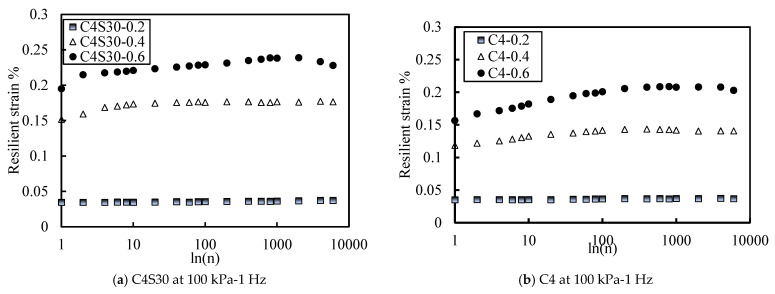
Curve of resilient strain with different CSR.

Figure 6a,b show the curves of elastic strain versus number of vibrations of C4S30 and C4 samples under three different CSRs when the effective confining pressure was 100 kPa. The variation range of the elastic strain of the C4S30 sample was about 0.03–0.24%, and that of the C4 sample was about 0.03–0.21%, which is much larger than that of the SAP content. In summary, a larger CSR leads to greater elastic strain, and the effect of CSR on elastic strain is much greater than that of SAP content.

**Figure 7:**
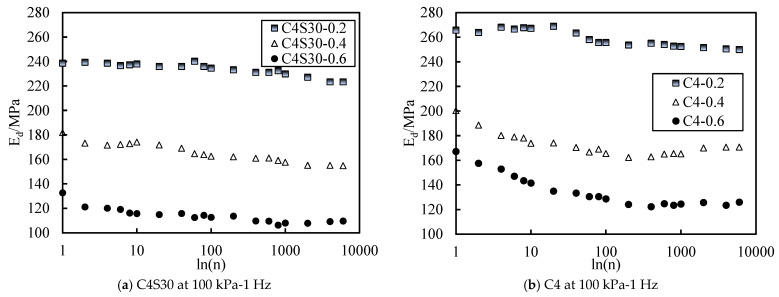
Curve of dynamic elastic modulus with different CSR.

Figure 7a,b are the curves of the dynamic elastic modulus versus the number of vibrations of C4S30 and C4 specimens, respectively under three different CSRs under the effective confining pressure of 100 kPa. It is not difficult to find that with the increase in cyclic strain ratio, the range of the dynamic elastic modulus of the soil samples increased. The C4S30 sample tended to be stable after about 200 vibrations, and the maximum variation range was 26 MPa. The C4 sample tended to be stable after about 600 vibrations, and the maximum variation range was 45 MPa.

In summary, with the increase in CSR, the elastic strain of the soil also increased, and the influence amplitude was greater than that of the SAP content. The dynamic elastic modulus of the soil decreased and the range of change became larger. The addition of SAP could narrow this range. This further indicates that SAP has a filling effect that cannot be ignored in soil.

#### 3.2.3. Resilient Strain at Different Vibration Frequencies

**Figure 8:**
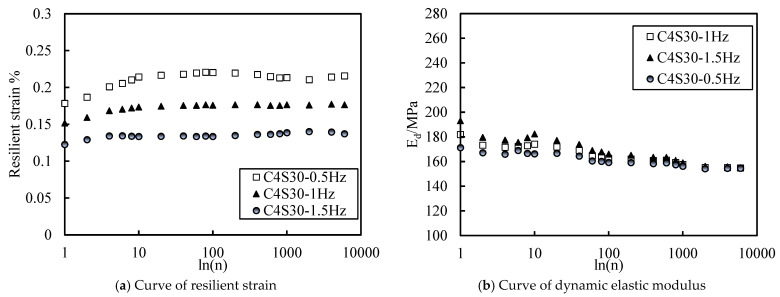
Curve of resilient strain behavior with different loading frequencies.

Taking the C4S30 soil sample as an example, the curves of elastic strain behavior affected by different vibration frequencies are plotted in Figure 8. It is not difficult to see from Figure 8a that the resilient strain of soil decreased with the increase in vibration frequency. This may be because the higher the vibration frequency, the shorter the reaction time of soil deformation, meaning the greater the deformation resistance of soil in unit time. Under the same CSR, the higher the vibration frequency was, the smaller the soil deformation was. Figure 8b shows the vibration frequency curve of the elastic modulus of C4S30. The three curves are close, and the fluctuation range was about 155–180 MPa. The fluctuation range was far less than the influence of SAP content and CSR, so it can be considered that the elastic modulus was not sensitive to the vibration frequency. This conclusion is also consistent with previous studies on dynamic characteristics of other types of soil [51,52].

### 3.3. Accumulated Plastic Strain of Soil

#### 3.3.1. Accumulated Plastic Strain under Different SAP Contents

**Figure 9:**
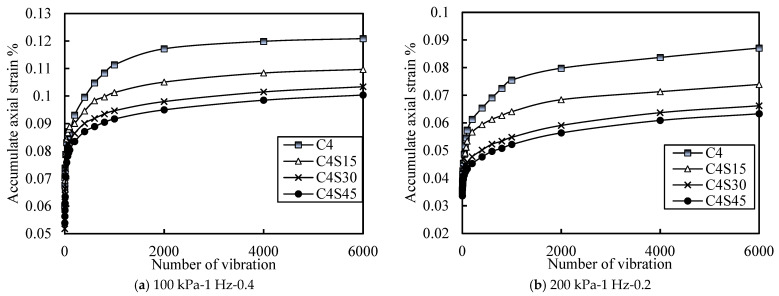
Curve of accumulated axial strain with different contents of SAP.

**Figure 10:**
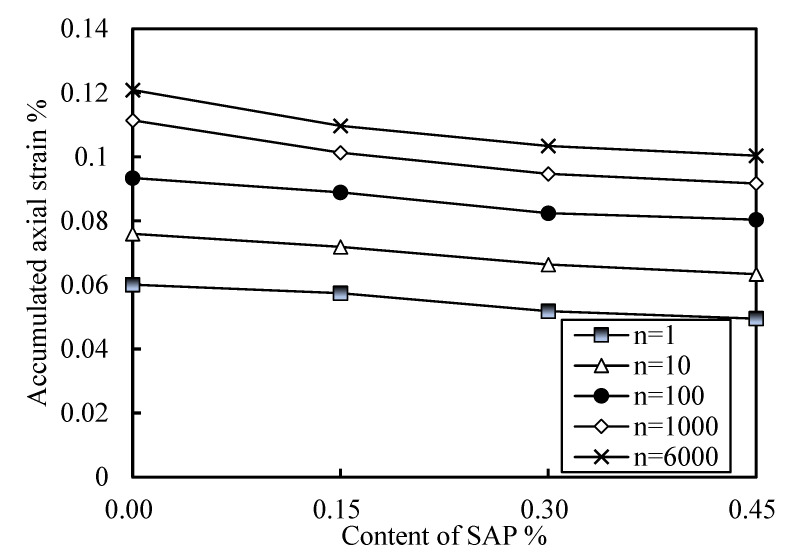
Relationship between accumulated axial strain and content of SAP.

Figure 9 shows the relationship curves of the cumulative axial strain of soil with vibrations under different SAP contents. Figure 9a shows an effective stress of 100 kPa, a vibration frequency of 1 Hz, and a CSR of 0.4. Figure 9b shows an effective stress of 200 kPa, a vibration frequency of 1 Hz, and a CSR of 0.2. By observing the whole, it is not difficult to see that each curve could be divided into two stages, namely, rapid growth stage and stable growth stage. Under the same effective stress and equal CSR, the accumulated axial deformation of soil increased with the increase in SAP content, indicating that the higher the SAP content is, the better the stability of the soil is. Furthermore, by comparing the trend and final stability value of each curve, it can be found that the soil samples without SAP entered the growth stability stage in a relatively lagged manner, and the growth rate of the stability value decreased with the increase in SAP content. In order to better illustrate this phenomenon, the accumulated axial strain under different SAP content is drawn in Figure 10 (effective stress of 100 kPa, vibration frequency of 1 Hz, CSR of 0.4, for example). With the increase in SAP content, the five lines under different cycles tended to be close, indicating that the influence of the cycles on cumulative strain decreased with the increase in SAP content. Combined with Figure 9 and Figure 10, it was found that most of the strain accumulations were completed in soil samples containing SAP before about 200 times, and most of the strain accumulations were completed in soil samples without SAP after 800 times.

In summary, SAP can make cemented soil enter the deformation stability stage faster and reduce the deformation caused by vibration, as well as improve the dynamic stability of cement soil. The higher the SAP content is, the better the improvement effect on cemented soil is. However, with the increase in SAP content, the improvement range is greatly reduced. Cement plays a major cementing role in soil, forming an “artificial cementation bond” [53,54]. Affected by vibration, the soil particles and the soil mass formed by cementation stagger each other and produce microcracks, which lead to the loss of the “artificial cementation bond” and a sharp increase in soil compressibility [53,55]. When SAP is added, SAP particles can fill the pores caused by the microcracks in time, so the soil is denser and there is no sharp increase in soil compressibility. Macroscopically, soil samples containing SAP enter the stage of deformation stable faster and have less accumulated axial strain. This phenomenon once again shows that SAP particles can play a good filling role in soil.

#### 3.3.2. Accumulated Plastic Strain under Different CSR

**Figure 11:**
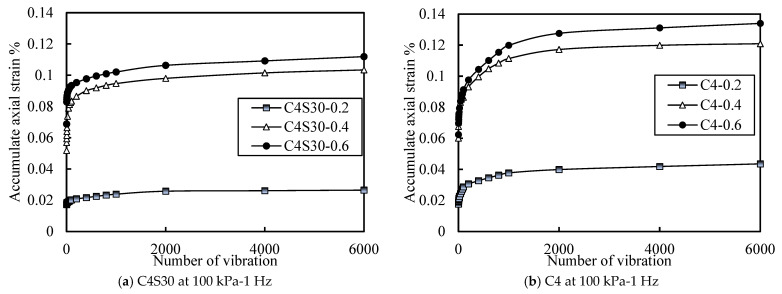
Curve of accumulated axial strain with different CSR.

**Figure 12:**
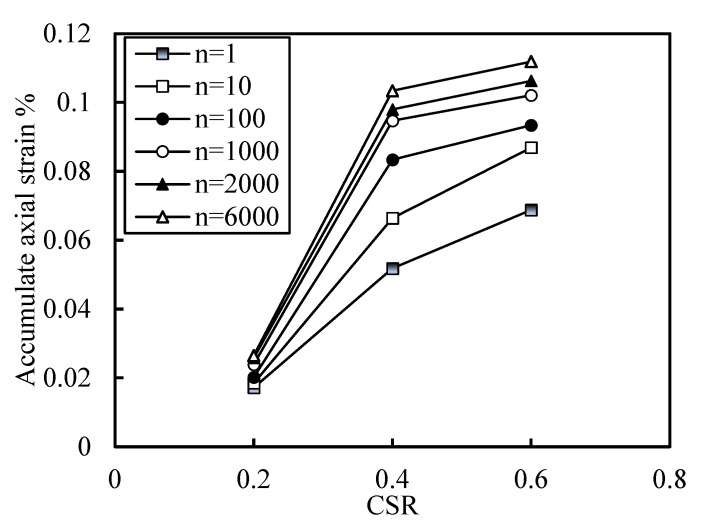
Relationship between accumulated axial strain and CSR (C4S30).

Figure 11a,b are the curves of accumulated axial strain versus vibration times for C4S30 and C4 specimens under different cyclic strain ratios, respectively. When the cyclic stress was small, the axial strain accumulation and increase rate were relatively small; when the CSR increased to a certain extent, the increase rate and size of axial strain accumulation had significant improvement. It is supposed that there may be a critical CSR. In order to show this more intuitively, the curve of CSR and cumulative axial deformation is drawn in Figure 12 (taking C4S30 as an example under the condition of an effective confining pressure of 100 kPa and a vibration frequency of 1 Hz). With the increase in CSR, the accumulated axial strain also increased. There was a critical CSR on the left side, and the increasing rate and magnitude of the accumulated axial strain were at a low level; on the right side, the increase rate and size of the cumulative axial strain of the soil samples were at a high level—much higher than that on the right side.

In summary, the accumulated axial strain of soil increases with the increase in CSR, and there is a critical CSR. When the critical CSR is exceeded, the cumulative axial strain of the soil increases significantly. It can be considered that the failure of soil depends on the critical CSR of soil, which can be further discussed in the future.

#### 3.3.3. Accumulated Plastic Strain at Different Vibration Frequencies

**Figure 13:**
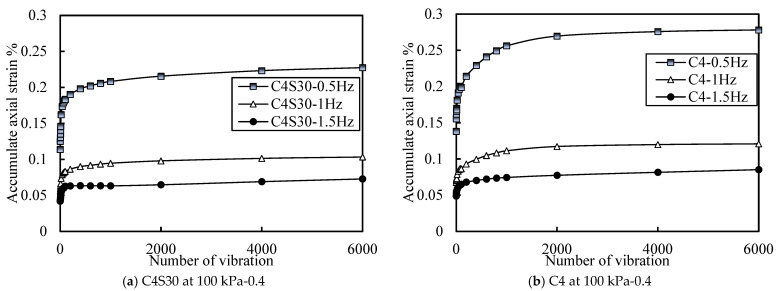
Curve of accumulated axial strain with different loading frequencies.

Taking the C4S30 and C4 samples as examples, the influence of dynamic load with different frequencies on the accumulated axial strain development of soil under an effective stress of 100 kPa and a CSR of 0.4 is drawn in Figure 13. Regardless of SAP, the accumulated axial strain of the soil decreased with the increase in vibration frequency. However, the accumulated axial strain of the soil at 1 Hz and 1.5 Hz was far less than that caused at 0.5 Hz. An important conclusion is drawn that attention should be paid to the influence of loading frequency on the dynamic characteristics of soil that is under the same stress level and cycles—if the loading frequency is low, the accumulated plastic strain of the soil will be large. Combined with the data from Figure 13, it is not difficult to see that the axial strain of the soil at 0.5 Hz was about 2.2–2.5 times that at 1 Hz and 1.5 Hz. Therefore, when subgrade backfill is used as soil around the subgrade and subway tunnel at the same time, it is necessary to pay attention to the frequency of the dynamic load under the two conditions, and the lower frequency should be taken as the reference standard.

This can be reasonably explained from two aspects. On the one hand is the energy transfer; on the other hand is the principle of effective stress. The deformation process of soil under traffic load is actually the process of vehicle or subway kinetic energy transferring to the soil, which partially offsets the internal structure energy of the soil. The continuous accumulation and transfer of kinetic energy breaks the internal energy balance of the soil, resulting in soil deformation. The more kinetic energy from road and tunnel operation is transferred, the greater the soil deformation is. Under the same stress level and cycle times, the lower the loading frequency, the slower the loading change, and the longer the loading time, the more conducive to the compaction of the soil. Therefore, the total energy transmitted to the soil is greater and the soil deformation is greater. Traffic load is related to cyclic vibration load. When the frequency is low, the soil has sufficient response time. According to the effective stress principle, this is the process of consolidation deformation. When the loading is too fast, the pore water pressure does not have enough time to increase completely and enters the unloading stage. Unless the loading frequency is high enough to destroy the basic structure of the soil sharply, the damage of high loading frequency is less serious than that of low loading frequency.

### 3.4. Effect of SAP on the Dynamic Strength of Cemented Soil

**Figure 14:**
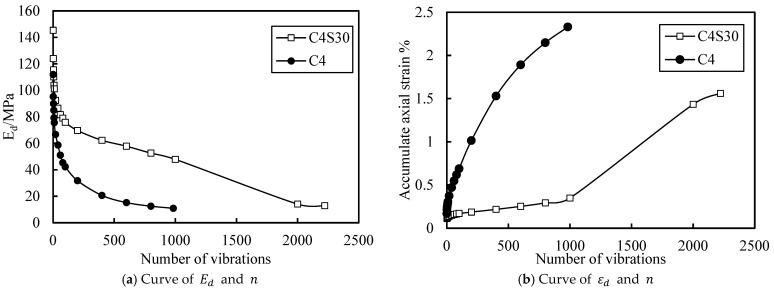
Curve of soil failure.

There were only two groups of samples with obvious failure in the cycle, which were samples C4 and C4S30 with an effective stress of 200 kPa, a CSR of 0.6, and a loading frequency of 1 Hz. The dynamic elastic modulus and accumulated axial strain of the two groups of samples are plotted in Figure 14a,b, respectively. At the beginning of the vibration, the axial stress of the cemented soil increased greatly and the dynamic elastic modulus decreased rapidly. The dynamic elastic modulus of the sample tended to be very small after 500 vibrations, and obvious cracks were observed on the sample. This phenomenon is consistent with previous studies on brittle failure of cement soil. At the initial stage of vibration, the dynamic elastic modulus of the C4S30 sample also decreased rapidly, but the initial dynamic elastic modulus was much higher than that of cemented soil and the decline rate was slower than that of cemented soil. The axial strain cumulative velocity was at a low level relative to the cemented soil, and it had the same change trend as the sample without failure. After about 1000 vibrations, the axial strain of the C4S30 sample began to increase rapidly, but its growth rate was slower than that of cement soil, and the decline rate of the dynamic elastic modulus did not change significantly. Finally, after about 2000 vibrations, the dynamic elastic modulus of the C4S30 sample tended to a small stable value and obvious cracks were observed in the sample. In summary, cement soil shows obvious brittle failure. The addition of SAP can significantly improve this behavior, increase the dynamic elastic modulus and failure cycle number of soil, and reduce the accumulated axial strain when the soil is damaged.

## 4. Conclusions

The purpose of this study was to explore the influence of SAP on the dynamic characteristics of cemented soil. The effects of SAP content, CSR, and loading frequency on the elastic strain, dynamic elastic modulus, and dynamic strain of cemented soil were studied. The specific conclusions are as follows:

(1) With the increase in SAP content, the overall deformation of cemented soil decreased and the cemented soil could enter the deformation stability stage more quickly, which improved the stability of the cemented soil. This also verified the filling effect of SAP in soil. However, with the increase in SAP content, the improvement effect on cemented soil deformation was greatly reduced.
(2) The addition of SAP improved the instantaneous deformation resistance and vibrations of cemented soil at a high stress ratio and reduced the accumulate axial strain of cemented soil, which effectively improved the brittle failure characteristics of cemented soil and improved its dynamic strength.
(3) The addition of SAP made cemented soil obtain better instantaneous deformation resistance under higher stress, but this ability was weakened under lower stress. SAP also had a similar effect on the dynamic elastic modulus of cemented soil. At low stress, even though the dynamic elastic modulus of soil containing SAP decreased, the reduction was limited and still far higher than the design requirements.
(4) The CSR had a significant effect on the elastic strain, dynamic elastic modulus, and cumulative axial strain of cemented soil and cemented soil containing SAP, whose influence exceeded that of SAP content. The larger the CSR was, the larger the elastic strain was and the smaller the dynamic elastic modulus was. The greater the CSR was, the greater the accumulated axial strain was.
(5) The loading frequency had little effect on the elastic strain and dynamic elastic modulus of cemented soil and cemented soil containing SAP, and there was no obvious trend. However, the loading frequency had a significant effect on the axial strain of cemented soil and cemented soil containing SAP, and the axial strain at 0.5 Hz was about 2.2–2.5 times that at 1 Hz and 1.5 Hz.

## Figures and Tables

**Table 1:** Basic physical properties of the excavated soil.

Natural Moisture Content (%)	Void Ratio	Optimum Water Content (%)	Liquid Limit (%)	Plastic Limit (%)	Plastic Index (%)	Organic Matter Content (%)
38.53	1.31	16.58	18.73	38.60	19.87	1.35

**Table 2:** Composition of the Portland cement and lime used in this study (%).

Binders	CaO	SiO_2_	Al_2_O_3_	Fe_2_O_3_	MgO	SO_3_	Other
Cement	58.95	23.4	6.3	3.98	4.85	1.5	1.02

**Table 3:** Test plan.

Symbol	Cement/%	SAP%	Effective Stress/kPa	CSR	Vibration Frequency/Hz
C4	4	-	100	0.2, 0.4, 0.6	1
C4S15	4	0.15	100	0.2, 0.4, 0.6	1
C4S30	4	0.30	100	0.2, 0.4, 0.6	1
C4S45	4	0.45	100	0.2, 0.4, 0.6	1
C4	4	-	200	0.2, 0.4, 0.6	1
C4S15	4	0.15	200	0.2, 0.4	1
C4S30	4	0.30	200	0.2, 0.4, 0.6	1
C4S45	4	0.45	200	0.2, 0.4	1
C4	4	-	100	0.4	0.5, 1.5
C4S30	4	0.30	100	0.4	0.5, 1.5

## Data Availability

All data included in this study are available upon request by contact with the corresponding author.
